# Intervening TNF-*α* via PPAR*γ* with Gegenqinlian Decoction in Experimental Nonalcoholic Fatty Liver Disease

**DOI:** 10.1155/2015/715638

**Published:** 2015-06-28

**Authors:** Yun-liang Wang, Li-juan Liu, Wei-han Zhao, Jun-xiang Li

**Affiliations:** Gastroenterology Department, Dongfang Hospital, Beijing University of Chinese Medicine, Beijing 100078, China

## Abstract

This paper is to explore the effect and mechanism of Gegenqinlian decoction on experimental nonalcoholic fatty liver disease (NAFLD) *in vivo* and *in vitro*. The final aim is to make clear whether Gegenqinlian decoction would impact NAFLD through improving PPAR*γ* to suppress inflammation and regulate lipid. The data in this research suggested that Gegenqinlian decoction is a potent way to manage NAFLD through improving PPAR*γ* to regulate lipid and suppress inflammation.

## 1. Background

Nonalcoholic fatty liver disease (NAFLD) is a kind of disease ranging from simple hepatic steatosis to nonalcoholic steatohepatitis (NASH) and irreversible cirrhosis [1]. The prevalence of NAFLD has risen rapidly in parallel with dramatic rise in obesity, diabetes [2, 3], hypertension, and dyslipidaemia and NAFLD is now regarded as the liver manifestation of metabolic syndrome (Mets). Up till now, the pathogenesis and progression of NAFLD are not yet fully understood; the proven therapeutic trails are still in improving [4, 5]. Some researchers approve the “2-hit” hypothesis on the NAFLD pathogenesis. Briefly, the “first hit” involves hepatic triglyceride accumulation, or steatosis. The “second hit” usually links inflammatory cytokines and oxidative stress and so forth. As concerned, hepatic triglyceride accumulation can occur as a result of increased fat synthesis, increased fat delivery, decreased fat export, and/or decreased fat oxidation [6]. Inflammatory cytokines, such as TNF-*α*, IL-6, and IL-8, are involved in the second hit stage, mediate steatohepatitis in patients with NAFLD [7], and can be regulated by PPAR*γ*, which is not only related to anti-inflammatory effects, but also close to improving insulin resistance [8].

Giving an effective therapeutic method for NAFLD/NASH to prevent future disease is of priority. Till now, intervention for NAFLD remains mainly through lifestyle modifications and nonpharmacological treatment. Hence, finding a new approach to NAFLD is in urgent need.

Chinese herbal medicine (CHM) has been traditionally used in China and other parts of Asian countries for thousands of years and is now spreading worldwide. A special and basic feature of Chinese medicine is the use of a formula containing several herbs (mixed as a cocktail) to ameliorate a series of abnormalities syndrome related to a certain disease. Herbal extracts contain multiple natural compounds that can aim to different targets via various pathological pathways involved in the disease, providing therapeutic effects via a spectrum of actions. Gegenqinlian decoction (GGQLD), a classical formula from* Treatise on Febrile Diseases, *is one of the well-known traditional Chinese medicines, which consists of* Kudzu root*,* Rhizoma coptidis, Scutellaria baicalensis *Georgi, and* Main licorice*. It is widely used to treat diarrhea. In previous studies, we have found that Gegenqinlian decoction can both improve diabetes [9, 10] and play an anti-inflammatory effect based on the compounds included [11–13], in which glucose and lipid metabolism disorder and inflammatory reaction play vital roles as in NAFLD. Hence, under the guidance of traditional Chinese medicine theory “same treatment for different diseases,” we propose a hypothesis that Gegenqinlian decoction could intervene and improve NAFLD by a multitargets solution.

## 2. Materials and Methods

### 2.1. Preparation of GGQLD and Rosiglitazone

GGQLD granules were provided by Pharmacy Department of Dongfang Hospital, Beijing University of Chinese Medicine (Beijing, China). The GGQLD granules contain equal weights of the ingredients of the Gegenqinlian formula:* Kudzu root *24 grams,* Rhizoma coptidis *9 grams*, Scutellaria baicalensis *Georgi 9 grams, and* Main licorice *6 grams. Rosiglitazone was purchased from Sigma, USA.

### 2.2. Animals and Treatment

Male Sprague-Dawley (SD) rats at 7 weeks of age were supplied by Vital River Laboratory Animal Technology Co. Ltd. (Beijing, China). All experimental procedures were approved by the Animal Ethics Committee of Beijing University of Chinese Medicine, following guidelines issued by Regulations of Beijing Laboratory Animal Management. SD rats were maintained on a 12 h light/dark cycle under constant temperature (22 ± 2°C) with* ad libitum *access to standard chow diet (*n* = 6) or a high-fat diet (HFD, 34% fat, 19% protein, and 47% carbohydrate by energy composition) (*n* = 6) for 8 weeks to induce NAFLD. The granules and rosiglitazone were dissolved in 100 mL of distilled water and kept at 2–8°C until use. The animals in GGQLD low dose (GGQLDL, 5.04 g/kg/day,* p.o.*, *n* = 6), GGQLD high dose (GGQLDH, 10.08 g/kg/day,* p.o.*, *n* = 6), and rosiglitazone (ROS, 20 mg/kg/day,* p.o.*, *n* = 6) were given at the beginning of HFD feeding; saline (10 mL/kg/day,* p.o.*, *n* = 6) was administrated to chow fed rats (chow, *n* = 6) as the model control. All of groups were given the drugs or saline for 8 weeks.

### 2.3. Cell Model Establishment and Intervention

HepG2 cells were cultured in Dulbecco's Modified Eagle's Medium (Gibco, USA) supplemented with 10% fetal bovine serum (FBS), penicillin (100 units/mL), and streptomycin (100 *μ*g/mL) at 37°C under 5% CO_2_, in a 95% humidified atmosphere. For the cell model establishing [14], palmitic and oleic acid 0.1 M stock solutions were prepared by dissolving free fat acid (FFA) in DMSO (with a final concentration less than 0.1%). The cells were exposed to a fresh mixture of exogenous 500 *μ*M FFA in molar ratio 1 : 2 palmitic : oleic for 24 h. Since albumin concentration is an important factor in determining the concentration of available FFA, 1%BSA for the final concentration was considered. For the intervention, different concentrations of GW9662 and PPAR*γ* selective agonist rosiglitazone [15] (Sigma, USA) were prepared and intervened together with FFA.

### 2.4. Determination of Metabolic Parameters: Liver Enzymes in Rats

At the end of treatment, animals were anesthetized using 4% chloral hydrate after a 12 h overnight fast; blood samples were collected from the abdominal aorta of rats. Fasting serum triglycerides (TGs), high-density lipoprotein cholesterol (HDL-C), and low-density lipoprotein cholesterol (LDL-C) were analyzed using Enzyme-linked immunoabsorbent assay (ELISA) (Bio Sino, Beijing, China). Fasting serum alanine aminotransferase (ALT) and aspartate aminotransferase (AST) were also determined by ELISA (Bio Sino, Beijing, China).

### 2.5. Histological Analysis in Rats

A fresh liver tissue was fixed with 10% formaldehyde solution. Paraffin sections were used to Haematoxylin and Eosin (H&E) staining (Zeping, Beijing, China). Frozen sections were stained with Oil Red O (Sigma-Aldrich). Both staining methods were used to investigate architecture of the liver and hepatic lipid droplets. Stained H&E and Oil Red O (ORO) slides were visualized with the Olympus microscope, and images were captured with Olympus digital camera (BX40, Beijing, China) using NIS Element SF 4.00.06 software (Beijing, China). For each group, liver samples from 3 to 5 rats were prepared and stained.

### 2.6. ELISA for Tumor Necrosis Factor-*α* (TNF-*α*) in Serum, Liver Tissue, and Cell Culture Media

After blood samples were collected, serum tumor necrosis factor-*α* (TNF-*α*) was tested by using rat ELISA kits (Boster Bio-Engineering, Wuhan, China).

Liver was collected and washed by ice-cold PBS and then stored at −80°C for subsequent histological and molecular assays.

Frozen liver tissue (0.5 g) was homogenized in 1 mL lysis buffer containing 150 mM NaCl, 1 mM PMSF, 10% glycerol, and the complete protease inhibitor cocktail (Roche, USA). The supernatant was collected for analysis of TNF-*α* using rat ELISA kits (Boster Bio-Engineering, Wuhan, China).

The TNF-*α* released in the culture media was determined by ELISA (Boster Bio-Engineering, Wuhan, China) according to manufacturer's instructions. Cells were cultured and treated as previously described. The culture media were collected after 24 h, possible contamination of cellular fractions was eliminated by centrifugation, and the test was performed in cell-free supernatant.

### 2.7. Western Blotting to Detect PPAR*γ*

Proteins in liver tissue homogenate were extracted using ice-cold tissue lysis buffer. Protein concentration was determined using a BCA protein assay kit. Samples were separated by 10% SDS-PAGE and transferred onto polyvinylidene difluoride membranes. The membranes were immunoblotted with primary antibodies that recognize PPAR*γ* (1 : 2000) and GAPDH (1 : 20000) (TDY Biotech, Beijing, China). Peroxidase-conjugated secondary antibodies and an ECL detection system were used according to routine methods. The intensities of the protein bands were analyzed using Gel-Pro 3.2 software. GAPDH protein was used as the internal control to normalize the protein loading.

### 2.8. Real-Time Polymerase Chain Reaction for PPAR*γ* mRNA Expression

Reverse transcription was performed with 1 *μ*g of total RNA per 12 *μ*L reaction using a standard cDNA Synthesis Kit (Takara, Japan). The real-time PCR primer sequences for target genes were as follows: PPAR*γ*, forward 5′-TACCACGGTTGATTTCTC-3′ and reverse 5′-GCTCTACTTTGATCGCACT-3′; GAPDH, forward 5′-TGGAGTCTACTGGCGTCTT-3′ and reverse 5′-TGTCATATTTCTCGTGGTTCA-3′ (CWbio, Beijing, China). For each real-time PCR, the typical thermal cycling conditions included an initial activation step at 95°C for 5 min, 45 cycles of amplification, and a final melting curve (55–95°C). For comparison of the PPAR*γ* mRNA levels, the cDNA concentrations were normalized with GAPDH PCR products. The data were analyzed using the ΔΔCt method.

### 2.9. Determination of Intracellular Fat Content: Oil Red O Staining and Quantifying

Cells were fixed and stained with Oil Red O and then washed by distilled water until the extracellular oil red is rinsed clean. The intracellular fats then trickle out after the rupture of cell membrane with 100% isopropyl alcohol. After shocks, OD values were read and analyzed by ELISA.

### 2.10. Data Analysis

All data are expressed as the mean ± SD unless otherwise indicated. Data were analyzed by using one-way ANOVA and a post hoc test. Differences were considered to be statistically significant at *P* < 0.05.

## 3. Results

### 3.1. Effect of Gegenqinlian Decoction on Lipid Metabolism and Liver Enzymes in HFD Rats

**Figure 1:**
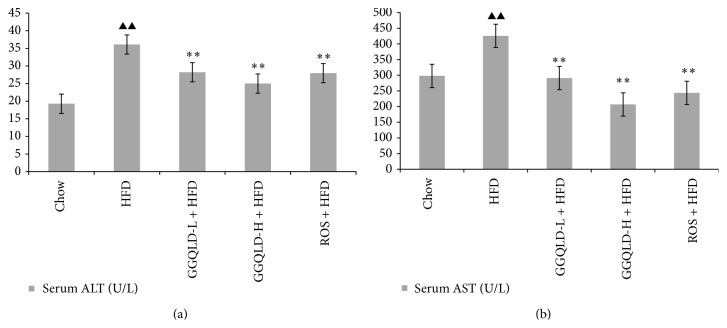
Effect of GGQLD on the concentration of ALT and AST. (a) ALT levels in serum; (b) AST levels in serum.* In vivo* study, data are means ± SD; *n* = 6 rats/group. ^▲^ *P* < 0.05, ^▲▲^ *P* < 0.01 versus chow; ^*∗*^ *P* < 0.05, ^*∗∗*^ *P* < 0.01 versus HFD.

**Figure 2:**
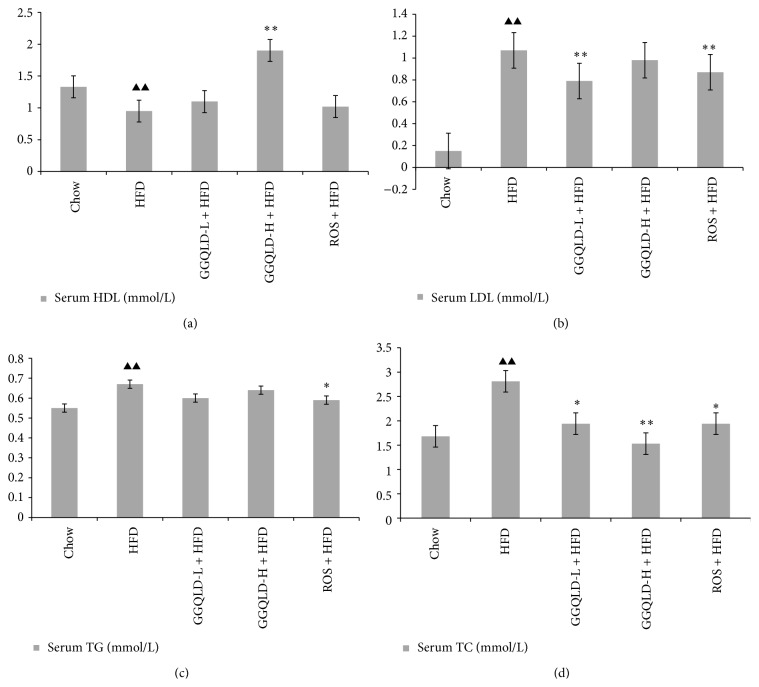
Effect of GGQLD on the concentration of triglyceride (TG), total cholesterol (TC), high-density lipoprotein (HDL), and low-density lipoprotein (LDL). (a) HDL levels in serum; (b) LDL levels in serum; (c) TG levels in serum; (d) TC levels in serum. In the* in vivo* study, data are means ± SD; *n* = 6 rats/group.  ^▲^ *P* < 0.05, ^▲▲^ *P* < 0.01 versus chow; ^*∗*^ *P* < 0.05, ^*∗∗*^ *P* < 0.01 versus HFD.

All animals tolerated the experimental procedures well, and no deaths occurred during the study. Serum ALT [(36.10 ± 3.92 U/L)] and AST [(425.85 ± 13.84 U/L)] concentrations in HFD fed rats were significantly higher than those in chow fed rats [(19.27 ± 3.36) U/L in ALT, (298.10 ± 5.13) U/L in AST, *P* < 0.01, resp.]. Both the GGQLDL [(28.20 ± 3.01) U/L in ALT, (291.24 ± 22.36) in AST] and GGQLDH [(24.98 ± 1.55) U/L in ALT, (206.68 ± 20.48) in AST] treatments significantly attenuated the elevated ALT and AST (*P* < 0.01), together with rosiglitazone [(27.98 ± 3.14) U/L in ALT, (243.48 ± 26.52) in AST, versus HFD, *P* < 0.01, resp.] (Figure 1). HDL-C levels rose in GGQLDH [(1.90 ± 0.10 mmol/L)] group compared to HFD model group [(0.95 ± 0.04) mmol/L, *P* < 0.01]. Meanwhile, GGQLDH [(0.98 ± 0.15 mmol/L)] also decrease LDL-C concentrations, but the statistical significance was not achieved (*P* > 0.01), whereas GGQLDL [(0.79 ± 0.09 mmol/L)] and rosiglitazone [(0.87 ± 0.22 mmol/L)] groups significantly decreased LDL-C level compared to HFD model group [(1.07 ± 0.20 mmol/L, *P* < 0.01, resp.)]. Both GGQLDL [(1.94 ± 0.39 mmol/L)] and GGQLDH [(1.53 ± 0.11 mmol/L)] treatments significantly attenuated the elevated TC [(2.81 ± 0.79) mmol/L in HFD, *P* < 0.01, resp.)]. Rosiglitazone not only decreased serum TC [(1.94 ± 0.12 mmol/L)], but also TG [(0.59 ± 0.02) mmol/L versus (0.67 ± 0.04) mmol/L in HFD, *P* < 0.01] (Figure 2).

### 3.2. GGQLD Improved Histopathology in Rats

**Figure 3:**
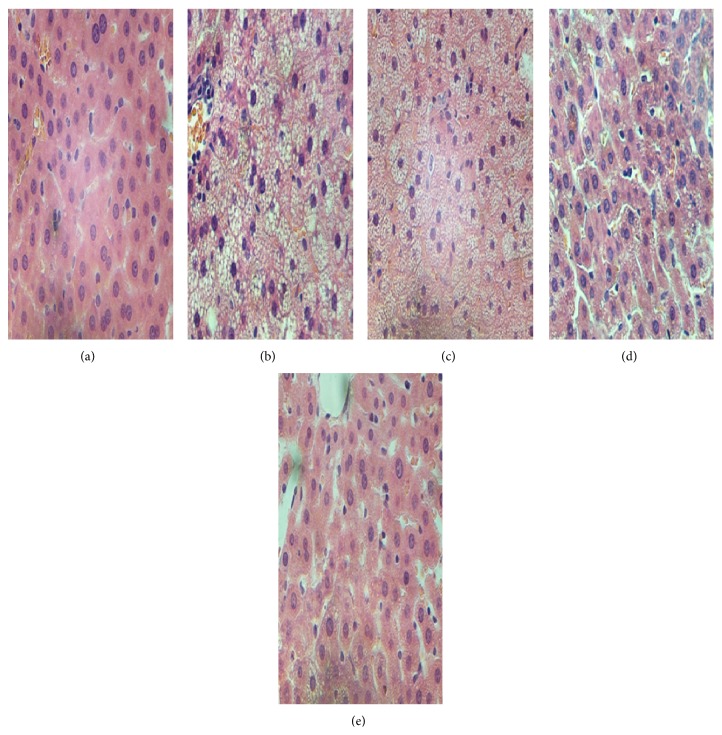
The results of paraffin section and H&E staining* in vivo*, ×400. (a) Chow fed rat, (b) HFD model rats, (c) GGQLDL treatment rats, (d) GGQLDH treatment rats, and (e) ROS treatment rats.

The photomicrographs of the H&E stain showed that HFD feeding increased hepatic fat deposits, evidenced by the majority of the hepatocytes of HFD rats that were distended by fat in comparison to the chow group (Figures 3(b) and 3(a)). The images of H&E stain also displayed steatosis and ballooning degeneration and evident infiltration with inflammatory cells in the intercellular substance, causing conspicuous swelling of the cell and cytoplasmic vacuolation as shown in Figure 3(b). The treatment of HFD rats with GGQLDs and rosiglitazone reduced fat deposits in liver (Figures 3(c), 3(d), and 3(e)) and the GGQLDs showed histological features similar to the chow group with little steatosis as shown in Figures 3(c) and 3(d).

**Figure 4:**
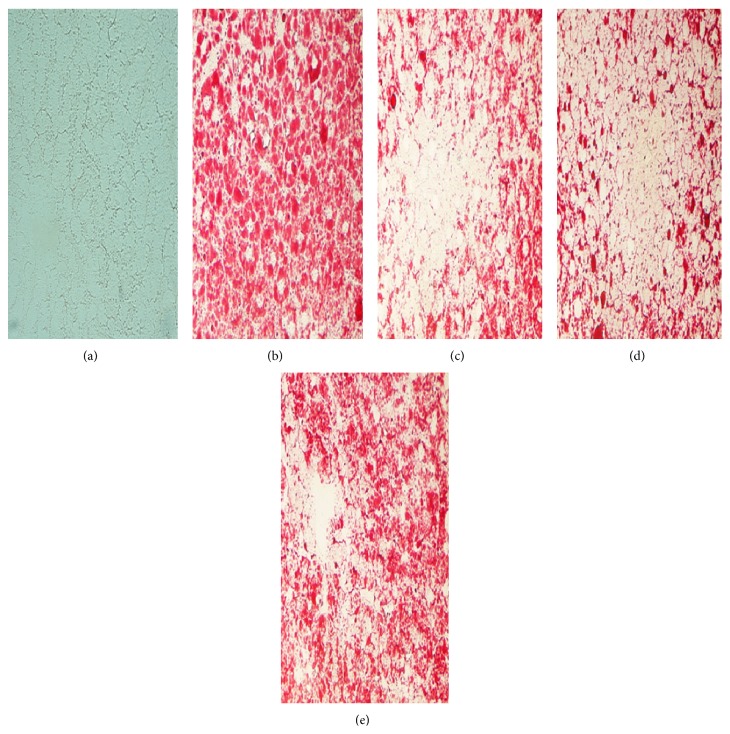
The results of frozen section and Oil Red O staining* in vivo*, ×400. The red blots show lipid drops in hepatocytes. (a) Chow fed rat, (b) HFD model rats, (c) GGQLDL treatment rats, (d) GGQLDH treatment rats, and (e) ROS treatment rats.

Oil Red O staining on frozen liver sections showed few lipid droplets were detected in the liver sections from the chow group (Figure 4(a)). Compared with that of HFD fed model rats (Figure 4(b)), both GGQLDs and rosiglitazone remarkably restrained lipid droplets deposited in hepatocytes (Figures 4(c), 4(d), and 4(e)).

### 3.3. Effects of GGQLD on Serum and Liver TNF-*α*

**Figure 5:**
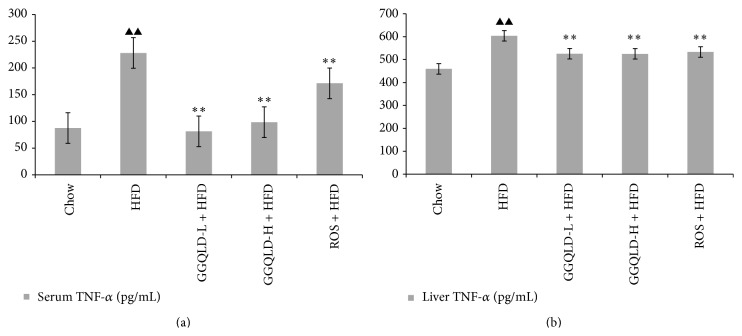
The effect of GGQLD on TNF-*α* level in serum and liver tissue. (a) TNF-*α* levels in serum; (b) TNF-*α* levels in liver tissue. In the* in vivo* study, data are means ± SD; *n* = 6 rats/group. ^▲^ *P* < 0.05, ^▲▲^ *P* < 0.01 versus chow; ^*∗*^ *P* < 0.05, ^*∗∗*^ *P* < 0.01 versus HFD.

Serum and liver TNF-*α* were determined in chow fed rats and HFD rats treated with saline, GGQLDL, GGQLDH, or rosiglitazone, respectively. As shown in Figures 5(a) and 5(b), HFD feeding significantly increased TNF-*α* compared with chow fed rats [(228.05 ± 26.85) pg/mL in HFD versus (87.59 ± 53.23) pg/mL in chow, in serum, *P* < 0.01; (603.68 ± 45.57) pg/mL in HFD versus (459.50 ± 12.53) pg/mL in chow, in liver, *P* < 0.01]. GGQLDL [(81.41 ± 15.39) pg/mL in serum, (525.55 ± 19.77) pg/mL in liver], GGQLDH [(98.42 ± 26.03) pg/mL in serum, (525.05 ± 58.87) pg/mL in liver], and rosiglitazone [(171.23 ± 18.63) pg/mL in serum, (533.30 ± 24.14) pg/mL in liver] were capable of significantly inhibiting HFD-induced elevated TNF-*α* both in serum and in liver (versus HFD, *P* < 0.01, resp.) (Figures 5(a) and 5(b)).

### 3.4. GGQLD Regulated PPAR*γ* Expression in HFD-Induced NAFLD Rats

**Figure 6:**
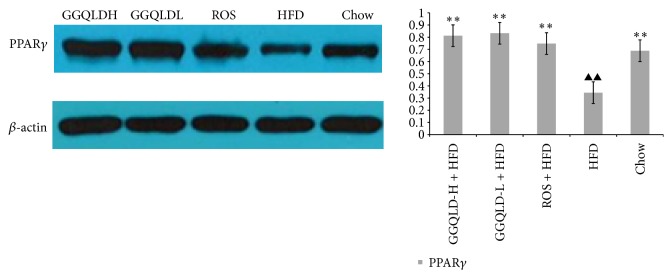
Western blotting for PPAR*γ* in liver tissue. In the* in vivo* study, data are means ± SD; *n* = 6 rats/group. ^▲^ *P* < 0.05, ^▲▲^ *P* < 0.01 versus chow; ^*∗*^ *P* < 0.05, ^*∗∗*^ *P* < 0.01 versus HFD.

We investigated whether GGQLD had a regulatory effect on PPAR*γ* expression. As shown in Figure 6, the PPAR*γ* level was significantly decreased on HFD group (*P* < 0.01, versus chow group). More remarkably, GGQLDs and rosiglitazone produced a dramatically improved effect on PPAR*γ* level (versus HFD group, *P* < 0.01, resp., Figure 6).

### 3.5. Validation of GGQLD on PPAR*γ* Gene Expression with Real-Time PCR

**Figure 7:**
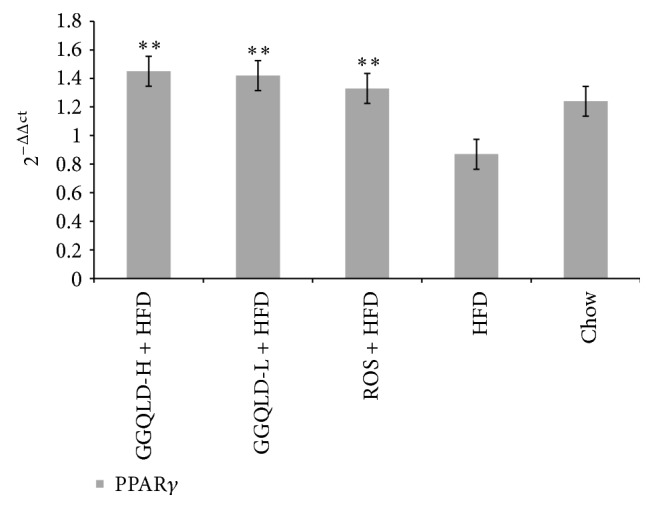
RT-PCR for PPAR*γ* expression in liver tissue. In the* in vivo* study, data are means ± SD; *n* = 6 rats/group. ^▲^ *P* < 0.05, ^▲▲^ *P* < 0.01 versus chow; ^*∗*^ *P* < 0.05, ^*∗∗*^ *P* < 0.01 versus HFD.

In order to confirm the results of effects of GGQLD on liver PPAR*γ* protein expression, the expression of PPAR*γ* gene was measured. The gene expression in HFD rat group decreased compared to chow group, but without a statistical significance (Figure 7). Remarkably, GGQLDs and rosiglitazone made a great contribution to increasing PPAR*γ* gene expression (versus HFD group, *P* < 0.01, resp., Figure 7).

### 3.6. Cell Release of TNF-*α* in the Culture Medium

**Figure 8:**
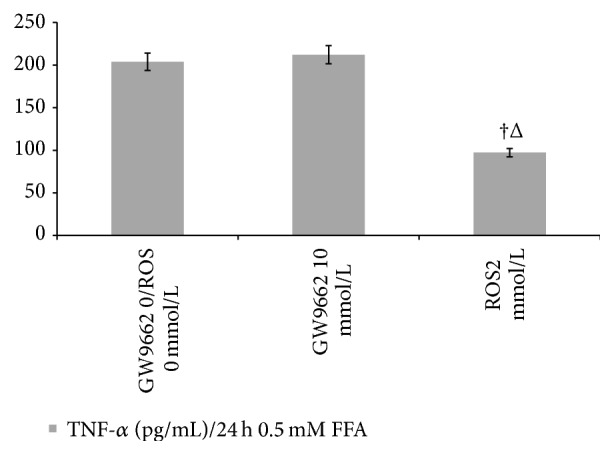
TNF-*α* level in supernatant in NAFLD cell model with GW9662 10 mmol/L and Ros 2 mmol/L. In the* in vitro* study, data are means ± SD; *n* = 5. ^†^ *P* < 0.05 versus model (GW9662 0/Ros 0 mmol/L); ^Δ^ *P* < 0.05 versus GW9662 10 mmol/L.

Compared with the model group [(203.83 ± 39.64) pg/mL], the production of TNF-*α* was evaluated in GW9662 10 mmol/L group [(212.17 ± 40.59) pg/mL, *P* > 0.05], while the TNF-*α* decreased in rosiglitazone 2 mmol/L group [(97.17 ± 2.93) pg/mL, *P* < 0.01] (Figure 8).

### 3.7. Determination of Intracellular Fat Content* In Vitro*

**Figure 9:**
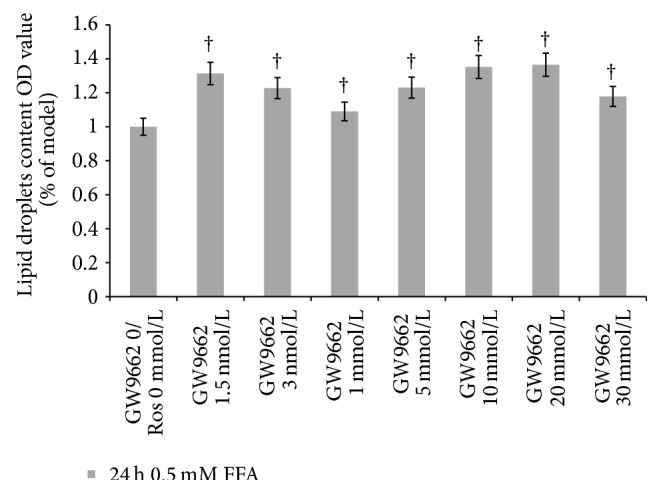
Lipid droplets content OD value (% of model) in NAFLD cell model. In the* in vitro* study, data are means ± SD; *n* = 5. ^†^ *P* < 0.05 versus model (GW9662 0/Ros 0 mmol/L); ^Δ^ *P* < 0.05 versus GW9662 10 mmol/L.

The intracellular lipid droplets content was analyzed by reading OD values; lipid droplets levels were increased significantly in groups with different concentrations of GW9662 compared with the model group (*P* < 0.05) (Figure 9).

## 4. Discussion

Nonalcoholic fatty liver disease (NAFLD), a multifactorial disorder with contribution of a variety of genetic and environmental factors, is considered to be closely associated with hepatic metabolic disorders, resulting in overaccumulation of fatty acids/triglycerides and cholesterol. The presence of steatosis is tightly associated with chronic hepatic inflammation [16], an effect in part mediated by activation of the Ik*κ*-b/NF-*κ*B signaling pathway. In murine models of high-fat diet induced steatosis, increased NF-*κ*B activity is associated with elevated hepatic expression of inflammatory cytokines such as TNF-*α* and activation of Kupffer cells [16]. TNF-*α*, a proinflammatory cytokine that is activated by the reactive oxygen species created by lipid peroxidation, not only promotes insulin resistance [17], but also mediates cholesterol and triglyceride metabolism [18]. It promotes necroinflammation, fibrogenesis, hepatic insulin resistance, and apoptosis [19]. Both serum and hepatic level of TNF-*α* are elevated in patients with NAFLD [20, 21], and levels correlate with histological severity [22]. Conversely, inhibition of TNF-*α* signaling improves IR and histological parameters of NAFLD [23, 24].

PPAR*γ* is one subtype of the three PPARs; the others are PPAR*β*/*δ*. PPAR*γ* agonists can promote adipose tissue to absorb and store FFAs and meanwhile inhibit liver fatty acid synthesis [25] via activating the AMP activated protein kinase (AMPK), of which the synthetic PPAR*γ* ligand (TZDS) has been used in the treatment of diabetes for many years. PPAR*γ* not only has anti-inflammatory effects, but also can effectively improve insulin resistance [8]. The activation of PPAR*γ* in immune system modulates inflammatory response [26]. In recent years, scholars have found that there were obvious changes in liver fat, increased adiponectin, and decreased TNF-*α*, IL-6, and resistin in patients with NAFLD after giving PPAR*γ* agonist treatment [27]. PPAR*γ* has been an important endogenous regulator and potential therapeutic target for nutritional NAFLD [28], of which the selective agonist [15] and an insulin-sensitizing agent [29] rosiglitazone has been a focus of treating NAFLD [30, 31].

High-fat diet induced animal model of NAFLD has been widely used to identify the pathogenesis and evaluate new treatments for NAFLD [32, 33]. In the present study, we have demonstrated that eight weeks of high-fat diet feeding induced fatty liver disease in SD rats, revealing key biochemical features of NAFLD, including increased elevation of hepatic enzymes, hyperinsulinaemia, hyperlipidaemia associated with increased TG accumulation in the liver, and histological changes containing steatosis, lobular and portal inflammation, and hepatocyte injury as ballooning, all about which reflected the characteristics of MetS [34]. Meanwhile, the histological abnormalities by H&E and ORO stains on the liver samples in the HFD rats of this study were consistent with existing reports [32, 35].

There are no pharmacological agents that have been approved for the treatment of NAFLD. Therefore, most clinical efforts have been directed focusing on the components of metabolic syndrome, namely, obesity, diabetes, dyslipidemia, and hypertension. Other interventions are directed at specific pathways potentially involved in the pathogenesis of NAFLD, such as insulin resistance, oxidative stress, and proinflammatory cytokines [36].

Chinese herbal medicine has been proven to play an anti-inflammatory effect in many fields of diseases [37, 38] and has a broad scope of drug safety. In the present study, treatment with GGQLD for 8 weeks has been proven to significantly normalize the liver hepatic aminotransferase (ALT and AST) to a level as nearly normal as chow control group. What is more, it was effective in impeding fat infiltration, which is evidenced by decreased hepatic TG contents and lipid droplets. Although the pathogenic mechanisms of NAELD are still under investigation, fat accumulation, especially triglycerides filtration into hepatocytes, is considered in the first step in development of NAFLD [4]. Hence, lipid accumulation plays a vital role with no doubt in NAFLD. The results from biochemistry and histology assays showed that GGQLDL and rosiglitazone produced an effect of reducing serums LDL-C and TC, respectively. Meanwhile, HDL-C level rose and TC decreased in GGQLDH group. What is more, histological stains showed GGQLDs and rosiglitazone decreased lipids droplets in hepatocytes and normalized steatosis in HFD rats. Overexpression of TNF-*α* has been identified and can mediate macrophage infiltration locally and at distant sites, such as liver. What is more, hepatic inflammation resulting from adipose proinflammatory cytokines does play an important role in the development of NAFLD [39]. The present study detected serum and liver TNF-*α* by biochemical method. The serum TNF-*α* level elevated in HFD group was shown in the present study, while decreasing in GGQLD and rosiglitazone groups. Besides, the expression of PPAR*γ* protein and gene was also measured. The protein and gene expression in HFD rats decreased compared to chow group. Remarkably, GGQLD made a great contribution to increasing PPAR*γ* protein and gene expression, with the equal effects in rosiglitazone group, which approved the effect of GGQLD on PPAR*γ*. In order to fully explore the PPAR*γ*/TNF-*α* pathway, we also clarified the relation between PPAR*γ* and TNF-*α* using PPAR*γ* antagonist GW9662 in a NAFLD cell model induced by free fat acid [14], of which the results showed that the production of TNF-*α* was evaluated in GW9662 10 mmol/L group, while the TNF-*α* decreased in rosiglitazone 2 mmol/L group, suggesting TNF-*α* is strongly related to PPAR*γ*. Meanwhile, lipid droplets levels were increased significantly in groups with different concentrations of GW9662 compared with the model group* in vitro*.

Numerous herbal products have been believed to have therapeutic benefit on NAFLD, such as silymarin (*milk thistle*), glycyrrhizin (*Main licorice*), curcumin (*Rhizoma curcumae longae*), berberine (*Rhizoma coptidis*), and puerarin (*Kudzu root*) [40–42], of which glycyrrhizin, berberine, and puerarin may give a great contribution to GGQLD on treating NAFLD. Puerarin, for example, has antioxidative and anti-inflammatory activity [43] and can exhibit therapeutic effect on NAFLD by antioxidation, lowering cholesterol, and improving leptin signal transduction [44, 45], while berberine improves glucose metabolism by inhibition of hepatic gluconeogenesis [46] and reduces methylation of the microsomal triglyceride transfer protein promoter in fatty liver [42].

Thus, based on the data in the present study, GGQLD had a positive intervention on NAFLD through improving lipid regulation and inflammation, which gave more lab data to support its clinical use. Because the medicine herbals in GGQLD have been used in traditional Chinese medicine (TCM) for thousand years, GGQLD has been considered for its safety and tolerability. This study explored that GGQLD is an optimal approach to NAFLD through managing lipid metabolic, inflammatory, and histological abnormalities via PPAR*γ*/TNF-*α* pathway. Further experiments would focus on systematic molecular mechanisms of GGQLD, using different blocking agents and advanced experimental techniques.

## Abbreviations

GGQLD: Gegenqinlian decoction
NAFLD: Nonalcoholic fatty liver disease
NASH: Nonalcoholic steatohepatitis
H&E: Haematoxylin and Eosin
ORO: Oil Red O
ROS: Rosiglitazone
TNF-*α*: Tumor necrosis factor-*α*
PPAR*γ*: Peroxisome proliferator activated receptor *γ*.
